# The patient safety practices of emergency medical teams in disaster zones: a systematic analysis

**DOI:** 10.1136/bmjgh-2019-001889

**Published:** 2019-11-14

**Authors:** Ussamah El-khani, Hutan Ashrafian, Shahnawaz Rasheed, Harald Veen, Ammar Darwish, David Nott, Ara Darzi

**Affiliations:** 1 Institute of Global Health Innovation, Imperial College London, London, UK; 2 The Royal Marsden Hospital NHS Trust, London, UK; 3 Former Chief Surgeon, International Committee of the Red Cross, Geneva, Switzerland; 4 Manchester Royal Infirmary, Manchester, UK

**Keywords:** health policy, health systems evaluation, systematic review, health services research, public health

## Abstract

**Introduction:**

Disaster zone medical relief has been criticised for poor quality care, lack of standardisation and accountability. Traditional patient safety practices of emergency medical teams (EMTs) in disaster zones were not well understood. Improving the quality of healthcare in disaster zones has gained importance within global health policy. Ascertaining patient safety practices of EMTs in disaster zones may identify areas of practice that can be improved.

**Methods:**

A systematic search of OvidSP, Embase and Medline databases; key journals of interest; key grey literature texts; the databases of the WHO, Médecins Sans Frontieres and the International Committee of the Red Cross; and Google Scholar was performed. Descriptive studies, case reports, case series, prospective trials and opinion pieces were included with no limitation on date or language of publication.

**Results:**

There were 9685 records, evenly distributed between the peer-reviewed and grey literature. Of these, 30 studies and 9 grey literature texts met the inclusion criteria and underwent qualitative synthesis. From these articles, 302 patient safety statements were extracted. Thematic analysis categorised these statements into 84 themes (total frequency 632). The most frequent themes were limb injury (9%), medical records (5.4%), surgery decision-making (4.6%), medicines safety (4.4%) and protocol (4.4%).

**Conclusion:**

Patient safety practices of EMTs in disaster zones are weighted toward acute clinical care, particularly surgery. The management of non-communicable disease is under-represented. There is widespread recognition of the need to improve medical record-keeping. High-quality data and institutional level patient safety practices are lacking. There is no consensus on disaster zone-specific performance indicators. These deficiencies represent opportunities to improve patient safety in disaster zones.

**Key questions**

**What is already known?**

- Communities affected by disaster, predominantly in low/middle-income countries, often rely on the humanitarian efforts of emergency medical teams (EMTs) to provide clinical care and support local health systems.
- Current EMT safety guidelines tend to focus on capacity requirements or management of specific surgical presentations, and are therefore not as broad as safety guidelines found in established high-income country.

**What are the new findings?**

- The initial search of 9685 records identified 30 peer-reviewed papers and 9 key grey literature texts which generated 302 individual patient safety statements that were categorised using the International Patient Safety Classification (ICPS) framework as well as our own thematic analysis.
- Most patient safety practices were associated with acute clinical care, especially surgery, with a noticeable lack of standardisation of medical record-keeping, patient safety practices relating to institutional monitoring, non-communicable disease (NCD) management and disaster zone-specific indicators.

**What do the new findings imply?**

- The lack of compatibility of the ICPS framework to the dataset in this review suggests the need for a disaster-specific patient safety framework and language.
- There is a need for EMT medical record standardisation, disaster zone-specific patient safety indicators, expansion of clinical guidelines to incorporate safe NCD management and improvement in patient safety culture.

## Introduction

Natural and man-made disasters represent a significant global health burden, particularly in low/middle income countries (LMICs). Most disasters and their associated mortality occur in LMIC, yet there is a paucity of disaster-related research from these countries.^1^ Projected rises in global urbanisation, population growth and competition over natural resources are expected to lead to further natural and man-made disasters.^2^ Vulnerable populations struck by disaster have often relied on the humanitarian efforts of emergency medical teams (EMTs) to provide medical care and support local health systems. While EMTs have largely focused on trauma and surgery, they have also been deployed in outbreak responses, such as the Ebola crisis.^3^ Historically, there has been a lack of standardisation of care and coordination in medical humanitarian responses and between EMTs.^4^

The incidence of patient harm in the UK was thought to be 10% of all hospitalised patients.^5 6^ Though there is a lack of patient safety data from LMIC for comparison, the available evidence suggests that by any patient safety indicator (PSI), outcomes are worse in LMIC compared with high-income country (HIC) to the extent that medical harm alone is thought to be the 14th most common cause of morbidity in LMIC.^7–11^ Specifically within disaster zones, predominantly a LMIC phenomenon, there is even less available data on patient safety outcomes.

Due to concerns over the clinical competence and lack of standardisation, coordination and accountability of some EMTs,^12^ the WHO launched the Global EMT initiative in 2010 and the Global EMT Registry in 2015. This allows EMTs to apply for classification status by demonstrating adherence to accepted guidelines and quality of healthcare. Registered EMTs are then deployed and coordinated via the host government and the WHO during a disaster to ensure quality of care.^3^

While guidelines^13 14^ have been available for many years, they provide generic prehospital and hospital clinical guidance on a cluster of specific conditions, or stipulate capacity requirements for delivery of healthcare in the predeployment and deployment phases. In HIC and in non-disaster settings, there are PSIs to evaluate an organisation’s patient safety culture.^13^ This level of detail and monitoring is lacking in the humanitarian sector, and therefore patient safety vigilance within EMTs is currently not well understood. Accurate data recording is crucial to patient safety, as avoidable medical harm cannot be improved if it cannot be measured.^11^ EMTs should therefore have the means to record and act on clinical data to enable assessment of patient safety outcomes and medical harm. Recent evidence shows there is wide variation and lack of standardisation in medical record practices within EMTs.^15^ This hinders early warning notification, planning of follow-up care, transparency of outcomes and quality improvement opportunities. As disasters predominantly occur in infrastructure and resource-poor areas, the patient safety challenges of EMTs are fundamentally different to those of a stable hospital setting in HIC. The validity of HIC non-disaster patient safety practices in an LMIC disaster setting has not been established and warrants further study.

The aim of this paper is to identify the pattern of patient safety practices of EMTs in an LMIC disaster zone. This will help identify which areas of EMT practice are currently adequately performed and which areas require improvement. By identifying where patient safety practices can be improved during deployment to a disaster zone, it may be possible to enhance organisational, cultural, clinical and medical record outcomes in disaster zone medical relief.

## Methods

Any patient safety intervention used during deployment was considered for this review. This includes techniques to reduce medical error, improve diagnostic accuracy, enhance decision-making, PSI monitoring and follow-up methods. Any implementation or assessment of medical record-keeping in an EMT setting was also included.

### Patient and public involvement

Due to the nature of this subject, patient and public involvement was not possible.

### Inclusion and exclusion criteria

All descriptive studies, case reports, case series, prospective trials and opinion pieces were included. There were no limits on date of publication or language.

Studies were excluded if they did not involve patient safety practices, focused only on the predeployment phase or took place in a HIC.

### Search strategy

The following four search strategies were used for the OvidSp, Embase and Medline databases:

1. “Patient Safety” OR “Patient harm” OR “Medical error” OR “Patient safety indicator”.
2. “Emergency Medical Team” OR “Humanitarian” OR “Foreign medical team” OR “Crisis” OR “Disaster” OR “Austere” OR “Conflict”.
3. ICRC OR International Committee of the Red Cross OR Médecins Sans Frontieres.
4. “X Emergency Medical Team”, where X is the country of EMT origin, and, where given, the official title of the EMT. At the time of writing, there were 13 approved EMTs on the WHO Registry from 10 different countries.

Searches 1 to 4 were performed separately and the results compiled. The following searches (5 to 8) were used for all other databases, search engines and grey literature:

1. Key individual journal databases were searched manually using eight separate terms: patient safety, patient harm, medical error, medicines safety, disaster, humanitarian, foreign medical team and austere. Search terms were excluded if they occurred within the journal title. For example, ‘disaster’ was excluded when searching the journal of *Disaster Medicine and Public Health Preparedness*.
2. Online databases of the WHO, Médecins Sans Frontieres (MSF) and the International Committee of the Red Cross were searched separately using the term “patient safety”.
3. Grey literature key texts were identified prior to the search as articles of interest. They were screened directly for any examples of patient safety interventions or recommendations, and the results of which were added directly to the data synthesis.
4. A separate Google and Google Scholar search of all 13 EMTs listed on the WHO-EMT Registry (at the time of writing) was also performed in the following format: “X Emergency Medical Team”, where X is the country of EMT origin, and, where given, the official title of the EMT. The search was restricted to the first 40 pages of search engine results. Table 1 contains a summary of all data sources.

**Table 1:** Data sources for systematic review

Peer-reviewed literature database	OvidSP, Embase, Medline
Manual database search of journals of interest	*Disaster Medicine and Public Health Preparedness* *BMJ Quality and Safety* *Journal of International Humanitarian Action* *Journal of Patient Safety*
Online key player databases	WHO Médecins Sans Frontiers (MSF) International Committee of the Red Cross (ICRC)
Grey literature key texts	Classification and Minimum Standards for Foreign Medical Teams in Sudden Onset Disasters Sphere Handbook WHO Pan American Health Organisation (PAHO) Guidelines ICRC War Surgery Volume 1 ICRC War Surgery Volume 2 ICRC Management of Limb Injuries Registering and Monitoring of FMTs Minimum Technical Standards and Recommendations for Rehabilitation AusMAT National Critical Care and Trauma Response Centre—Guide 2011
Other	Google Google Scholar (first 40 pages of search results)

AusMAT, Australian emergency medical team; FMTs, foreign medical teams.

### Outcomes and data extraction

The primary outcome was any recommendation of a patient safety practice.

The secondary outcomes were the following: (1) recommendation to use any of the 21 Organisation for Economic Co-operation and Development (OECD) PSIs^16^ and (2) utilisation or recommendation of a medical record-keeping system or minimum dataset.

All primary and secondary outcomes were listed as single patient safety statements, for example, ‘the use of internal fixation for fracture stabilisation is contraindicated in a conflict zone’. Each statement was then classified as per the International Classification for Patient Safety (ICPS) framework^17^ and also classified by a separate thematic analysis.

All studies were graded as per the US Preventative Services Task Force Classification.^18^ This was chosen as it provides a clear score for each category of study, particularly those near the bottom of the hierarchy of evidence, of which many of the studies in this review were expected to be.

### ICPS framework

**Figure 1:**
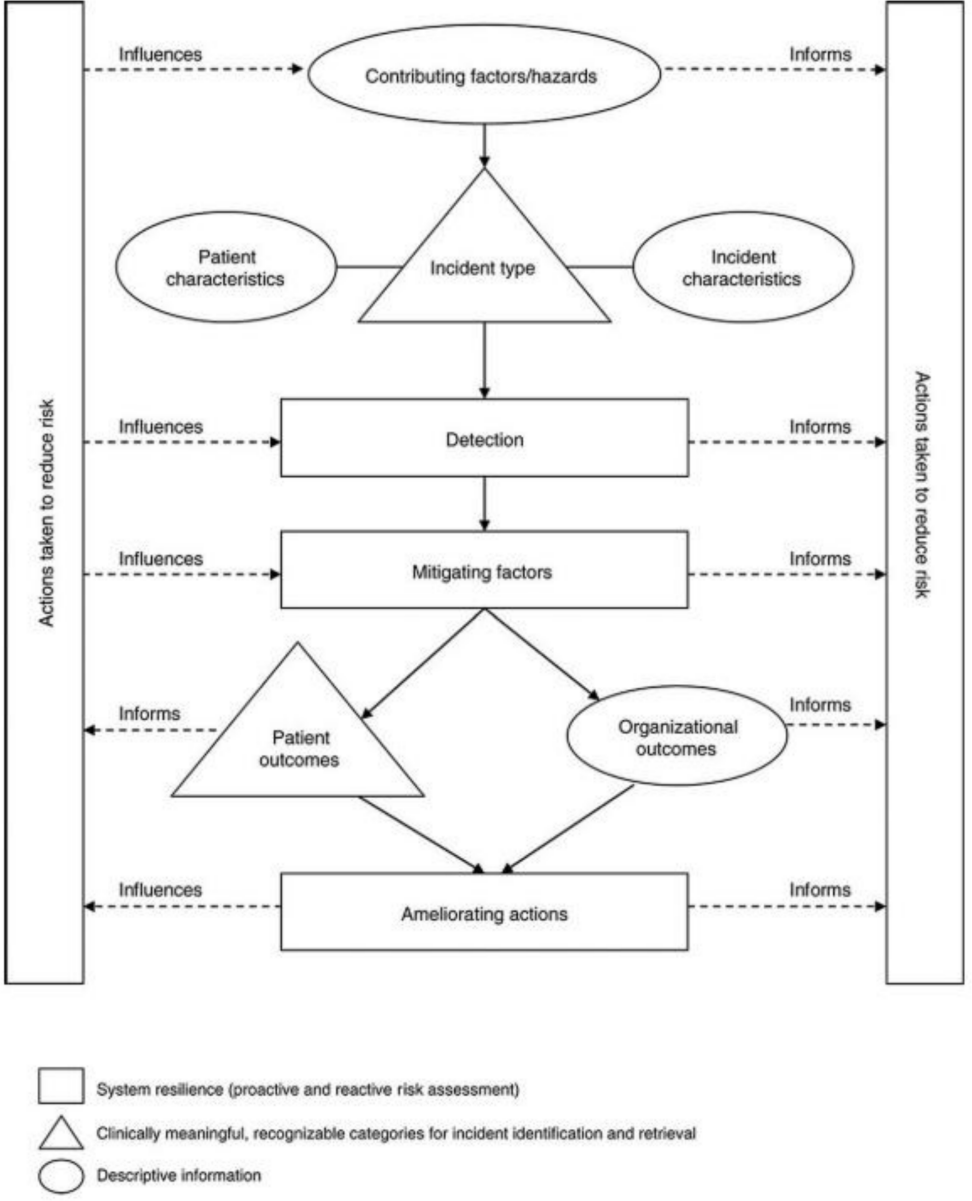
International Classification for Patient Safety framework; McElroy *et al*.^17^

The ICPS framework allows a patient safety incident to be classified and its position in the treatment pathway to be described using an algorithm^17^ (figure 1).

As the ICPS framework is designed to categorise past or existing incidents of harm, many of the patient safety statements were predicted to be syntactically incompatible with the framework. To overcome this limitation, each statement was categorised by the ICPS framework section ‘Actions to Reduce Risk’, which the authors felt was the section of the framework that was most compatible with the dataset. Where this was not possible, the statement would then be classified in order of preference by ‘Incident Type’, then ‘Detection’, then ‘Mitigating Factors’ and finally ‘Ameliorating Actions’, until the best description was applied to each statement. For conciseness, only the one root that was thought to most accurately reflect the patient safety statement would be selected. For example, the statement ‘The referral document must be included with the patient’ would be classified under ‘Medical Records’ rather than the full root description of ‘Incident Type—Documentation—Problem—Document Missing or Unavailable’. A patient safety statement could be assigned more than one ICPS framework descriptor.

### Thematic analysis

The statements were also thematically analysed in a process separate to the ICPS framework. The OECD PSIs^16^ and the findings of the articles generated from the systematic search guided the level of detail when assigning codes. A code was applied to each patient safety statement followed by a second round of elimination and categorisation. A patient safety statement could be assigned more than one category. The codes were assigned by author UE and cross-checked by HA.

### Role of the funding source

The funder of the study had no role in the study design, data collection, data analysis, data interpretation or writing of the report. The corresponding author had full access to all the data in the study and had final responsibility for the decision to submit for publication.

## Results

**Figure 2:**
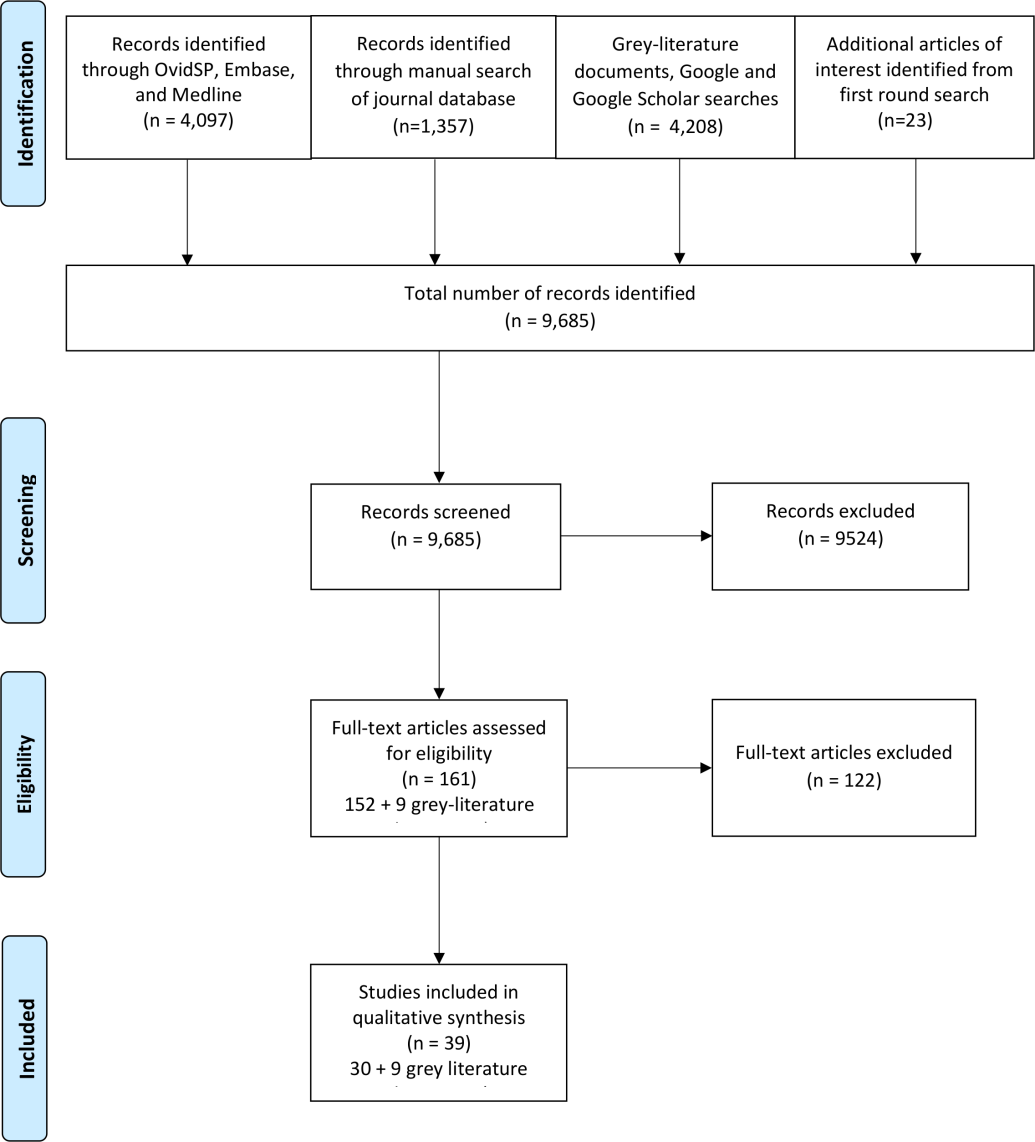
PRISMA (Preferred Reporting Items for Systematic Reviews and Meta-Analyses) flow diagram of patient safety practices of emergency medical teams in disaster zones.

The total number of records identified in the peer-reviewed literature was 5454. The grey literature^13 14 19–25^ and searches of the online databases of the WHO, MSF and ICRC generated 3399 records. The total number of records screened was 9685, most of which were excluded as they were not related to EMT practice. A further 122 were excluded as they either related to EMT practice in the predeployment phase, took place in a HIC or did not relate to patient safety practices. The remaining 39 records comprised 30 peer-reviewed papers^15 26–54^ and 9 grey literature documents to be included in the data synthesis.^13 14 19–25^ The 30 peer-reviewed papers generated 82 patient safety statements, and the grey literature generated 220 patient safety statements. The total number of patient safety statements was 302 (figure 2).

### Quality of evidence

The overall quality of evidence of the 30 peer-reviewed papers was poor, with only one study^39^ categorised as level II-2 evidence as per the US Preventive Services Task Force classification. The remaining 29 studies comprised level II-3 and level III (table 2).

**Table 2:** Quality of evidence of the peer-reviewed literature

Author	Year	Disaster zone	EMT	Quality*	Theme
Salibi^26^	1975	Multiple	Not specified	III	Po
Rautio and Paavolainen^27^	1988	Afghanistan	ICRC	II-3	A, H, L, TA
Gertsch^28^	1987	Peshawar	ICRC	III	H, L, TA
Gosselin *et al*^29^	1993	Peshawar	ICRC	II-3	AI
Strada *et al*^30^	1993	Afghanistan	ICRC	II-3	TA
Sundin^31^	1995	Rwanda	ICRC	II-3	A, Abx, C, F, T, TA
Rowley^32^	1996	Afghanistan, Sudan	ICRC	II-3	F, L
Molde^33^	1998	Multiple	ICRC	III	F, L
du Mortier and Arpagaus^34^	2005	DRC	ICRC	II-3	I, M, S
Kanter *et al*^35^	2008	Multiple	Not specified	III	ICU, P
Chapman *et al*^36^	2008	Multiple	Not specified	III	Im
Clasper and Rowley^37^	2009	Sudan	ICRC	III	L
Ennis^38^	2010	Haiti	Project Medishare	III	W, Sc
Deering *et al*^39^	2011	Iraq	US MHS - CSH	II-2	In, Tr
Jawa *et al*^40^	2012	Haiti	Project Medishare	III	A, Abx, C, F, MR, TA
Sever *et al*^41^	2012	Multiple	Renal Disaster Relief Task Force	III	R
Zoraster and Burkle^42^	2013	Multiple	Not specified	III	MR
Asgary^43^	2013	Multiple	Not specified	III	Sp, T
Nessen *et al*^44^	2013	Afghanistan	US Military Forward Surgical Team	II-3	BPS
Einav *et al*^45^	2014	Multiple	Not specified	III	B, D, ICU, Ref
Elder *et al*^46^	2015	Multiple	MSF	III	Abx, B
Borovecki *et al*^47^	2015	Multiple	ICRC	III	PSC
Jafar *et al*^15^	2015	Multiple	Not specified	III	MR
Rau and Blake^48^	2015	Multiple	ICRC	III	D, MR, Ph
Ren *et al*^49^	2015	Philippines	Peace Ark Hospital Ship	II-3	Ra
Trudeau and Rothstein^50^	2016	Multiple	Multiple, including MSF	III	P
Cancedda *et al*^51^	2016	Sierra Leone	Partners in Health and Wellbody Alliance	III	E
Kuckelman *et al*^52^	2016	Afghanistan	US Military Forward Surgical Team	II-3	S
Bauhan and Veen^53^	2017	DRC	ICRC	II-3	F, L
Burkle^54^	2018	Multiple	Not specified	III	T

*Classified according to the US Preventive Services Taskforce. I. Properly powered and conducted RCT; well-conducted systematic review or meta-analysis of homogeneous RCTs. II-1. Well-designed controlled trial without randomisation. II-2. Well-designed cohort or case–control analysis study. II-3. Multiple time series, with or without the intervention; results from uncontrolled studies that yield results of large magnitude. III. Opinions of respected authorities, based on clinical experience; descriptive studies or case reports; reports of expert committees.

A, anaesthesia; Abx, antibiotics; AI, arterial injury; B, burns; BPS, blood products safety; C, chest; D, discharge planning; DRC, Democratic Republic of Congo; E, Ebola outbreak; EMT, emergency medical team; F, fracture; H, head injury; I, indicator; ICRC, International Committee of the Red Cross; ICU, intensive care unit; Im, immunisation; In, incident reporting; L, limb; M, medicines; MHS-CSH, Military Healthcare System—Combat Support Hospital; MR, medical records; MSF, Medicines Sans Frontieres; P, paediatric care; Ph, physiotherapy; Po, positioning of patient; PSC, patient safety culture; R, renal injury; Ra, radiology; RCT, randomised controlled trial; Ref, referral or second opinion; S, sharps disposal; Sc, scoring system; Sp, supervision; Su, surgery; T, triage; TA, thoracoabdominal; Tr, training.

### ICPS framework

There were 32 different ICPS codes with a total frequency of 333. The most common ICPS codes were clinical error treatment (88, 26.4%), protocol (31, 9.3%), medicines safety (26, 7.8%), general patient care and medical records (both 25, 7.5%) (table 3).

**Table 3:** Frequency of ICPS codes

ICPS code	Frequency (/333)	%
Clinical error—treatment	88	26.43
Protocol	31	9.309
Medicines safety	26	7.808
General patient care	25	7.508
Medical records	25	7.508
Equipment	14	4.204
Staff—capacity/quality/orientation	14	4.204
Detection of harm—audit/review	13	3.904
Clinical error—diagnosis/assessment	12	3.604
Infection control	11	3.303
Staff—training/supervision	10	3.003
Blood products safety	6	1.802
Patient safety culture	6	1.802
Staff—team management	6	1.802
Access to service	5	1.502
Clinical error—screening/prevention	4	1.201
Communication	4	1.201
Detection of harm—risk assessment	4	1.201
Tests/investigations	4	1.201
Transition of care	4	1.201
Consent	3	0.901
Nutrition	3	0.901
Patient education	3	0.901
Oxygen	2	0.601
Physical environment	2	0.601
Regulation	2	0.601
Administrative error	1	0.3
Clinical error—procedure/treatment	1	0.3
Complaint management	1	0.3
Detection of harm—systems monitoring	1	0.3
Leadership	1	0.3
Specimens handling	1	0.3

ICPS, International Patient Safety Classification.

### Thematic analysis

The thematic analysis generated 84 different themes with a total frequency of 632 (table 4). For the complete list of themes and their frequencies, please refer to online supplementary 1, table 5. The most common themes were limb injury (57, 9%), medical records (34, 5.4%), surgery decision-making (29, 4.6%), medicines safety and protocol (both 28, 4.4%).

10.1136/bmjgh-2019-001889.supp1 Supplementary data

**Table 4:** Fifteen most frequent thematic analysis codes

Code	Frequency (/632)	%
Limb injury	56	8.86
Medical records	34	5.38
Surgery—decision-making	29	4.59
Medicines safety	28	4.43
Protocol	28	4.43
Surgery—technique	28	4.43
Infectious disease	21	3.32
Abdominal injury	15	2.37
Physiological monitoring	15	2.37
Team	15	2.37
Training	15	2.37
Fracture	14	2.22
Wound care	14	2.22
Amputation	13	2.06
Indicator	13	2.06

Themes relating to direct clinical care, defined as a medically related interaction between a healthcare worker and patient, contributed 58 of the 84 themes (69%) with a total frequency of 407 (64.4%). Medicines safety was classified as a separate entity and comprised six separate themes: general medicines safety, blood product safety, antibiotics, immunisation, venous thromboembolism prophylaxis and intravenous fluids. In total, the frequency of medicines safety was 51 (8.1%).

Non-clinical descriptors, which relate to any non-clinical interaction between the healthcare worker or facility and a patient (such as medical record-keeping, availability of protocol, the use of performance indicators and team development) generated 20 separate themes with a total frequency of 174 (27.5%). Online supplementary 1, tables 6–8 summarise the most frequent clinical, non-clinical and medicines safety themes.

## Discussion

Overall, we identified that the majority of patient safety statements were clinical in nature, with a focus on aspects of surgery such as decision-making and technique. The most frequently occurring non-clinical themes were related to medical record-keeping and the availability of protocolisation. Most of the patient safety statements derived from the dataset in this review originated from the grey literature. The ICPS framework was not as compatible with the dataset compared with our thematic analysis.

The ICPS framework’s most frequent theme was ‘clinical error—treatment’ (26.4%). This broad description lacks the accuracy that the thematic analysis provided and therefore was not able to demonstrate that the nature of these clinical errors mainly related to the management of limb injury. Most of the clinical thematic codes were acute in nature, comprising mainly limb injury, surgery and its associated care, such as wound management, amputation, anaesthesia and skin grafts. The ICRC Surgical Database demonstrates that in a war setting, limb injury is the most frequently occurring wound, with an average of 65% of major wound presentations since World War I.^19^ As the ICRC texts featured heavily in our data in both volume and detail of clinical guidance, limb injury is thought to be over-represented in our results.^19–21^

As disaster zones includes non-war scenarios, it is possible that the incidence of limb injury presented to an EMT, which is expected to work in all sudden-onset disasters, may differ to those published from war zones. A recent review identified that the distribution of presentations to a medical facility in weather-related disasters for wounds and orthopaedic injuries ranged from 1.1%–54.6% to 0.8%–34.4%, respectively.^55^ Nonetheless, there is paucity of data to establish what is the true burden of patient safety failures in the management of limb injury across all disasters, and whether other aspects of disaster management within an EMT, such as non-communicable disease (NCD) management, require prioritisation.

The management of NCDs has gained prominence in LMIC global health policy. The burden of NCD in LMIC is accelerating, and of the 14 million preventable NCD-related deaths that occurred in the 30 to 70 year age group, 85% of those occurred in LMIC.^56 57^ While this is being addressed by the WHO Global NCD Action Plan,^56^ there is little guidance in the literature for EMTs to manage NCDs and chronic disease in a disaster setting. Presentations of acute exacerbations of chronic cardiovascular disease, stroke, uncontrolled diabetes and stunted weight in malnourished children increase during or following a variety of disasters.^55 58–61^ WHO-registered EMTs are categorised mainly on their surgical capacity and provision of specialty surgical services.^14^ In our review, a wide range of patient safety practices pertaining to clinical activity in the acute setting was demonstrated in the thematic analysis. Notably, NCD was under-represented with only two occurrences (0.32%), despite the increasing importance it appears to have in disaster presentations. This may prompt efforts to improve NCD management in EMTs, such as the provision of chronic disease guidelines, expansion of recommended essential lists to improve diagnosis and treatment, optimise medical record-keeping, patient education and follow-up. McDermott *et al* have demonstrated a model based on the experiences of the Australian EMT when managing patients with diabetes during Typhoon Haiyan in 2013, suggesting ways to expand the role of the EMT to manage NCDs in a disaster setting.^58^

All key texts in the grey literature search emphasised the importance of adequate medical record-keeping, with guidance on minimum data recommendations, injury classification systems, triage systems, documentation of consent, operation notes, follow-up documentation and dissemination of data for institutional and regional monitoring.^13 14 19–25^ Of the non-clinical codes in the thematic analysis, 20% were related to medical record-keeping. Adequate medical record-keeping is essential in patient safety,^15^ and this has especially been recognised in disaster response due to the lack of standardisation of EMT recording practices.^15 62^ The WHO-EMT Registry programme stresses the importance of minimum datasets and sharing of accurate medical records. It does not mandate the use of a particular electronic or written medical record system. Inconsistent use of language can compromise understanding of patient safety, hence the ICPS framework was designed to be a standardised language compatible with existing WHO classifications.^63^

It became apparent that the ICPS framework terminology was not fully compatible with the data in this review due to syntax mismatch. This then prompted the thematic analysis. Online supplementary 1, table 6 shows a comparison of the 10 most frequent ICPS and thematic analysis themes. While there is some overlap, there were obvious differences in the type of theme and their frequencies. These differences support the suggestion that current data recording practices in EMTs are not conducive to evaluation with the ICPS framework.

Freely accessible platforms to improve reporting from disaster zones have been available for decades.^64 65^ Differences in reporting styles, preference to publish in established journals, time pressures and limitations in technology may partially explain the poor uptake of these systems. Mills *et al* highlight the challenges in creating a comprehensive humanitarian relief database which include creating a culture of participation, minimising the threat to agencies and staff, and encouraging academics to release their findings prior to journal publication.^62^ Therefore, challenges to standardised, accurate and accessible data from the field span the entire hierarchy of EMT stakeholders; from governmental support right through to the patient safety culture of front-line workers and provision of the required technology and training. We believe all these factors need to be addressed to incentivise the development and adoption of a standardised open source electronic medical recording system in disaster zones.

Of the non-clinical themes in the thematic analysis, there was a preponderance to patient safety practices at a clinical team level as opposed to a higher managerial or institutional level. Protocol, team, training, capacity, supervision and debrief comprised 42.5% of non-clinical descriptors compared with the 8.6% of institutional monitoring, incident report and complaints management. Patient safety practices in established HIC settings encourage participation and leadership from hospital managers in addition to clinicians and patients.^66^ Organisational-related patient safety leadership in an EMT appears to be bottom-heavy and less manager driven. This may be related to the nature of EMTs often comprising smaller teams with a flatter hierarchy. This provides an opportunity to improve patient safety practices by ensuring clinical teams are adequately supported by the EMT management and patient safety culture is allowed to develop across the entire EMT hierarchy.

There were 11 categories of performance indicators in the reviewed literature (online supplementary 1, table 9), 28% of which related to medical record-keeping. Of the 21 OECD indicators, only ‘transfusion reaction’ was encountered in this review.^16^ This suggests that disaster response requires its own unique indicators not currently offered by HIC patient safety practices. Conversely, some of the indicators suggested in the review data, such as malaria fatality rates, drug donation guideline compliance and displaced population measles vaccine coverage, are unlikely to feature significantly in HICs. A WHO report recommends reporting on the health impact of disasters at a national level as well as disaster-related deaths.^67^ These indicators may provide useful epidemiological data to guide resource allocation; however, they do not allow the safety practices of individual EMTs to be assessed. Just as the ICPS framework may lack validity in a disaster setting due to poor data capture and language incompatibility, too many of the established patient safety indicators are currently in use.

The grey literature dominated the dataset in this review. This can be partly explained by the relative infancy of the concept of delivering quality of healthcare in disaster zones, whereas patient safety leadership in HIC has been present since the turn of the century. This suggests that the evidence to support current safety practices in EMTs does not currently exist. The poor quality of evidence in this review and its heterogeneity, which precluded a quantitative analysis, are obvious limitations of this paper. Another limitation was the exclusion of data that originated from HIC disasters. As mortality from disasters is proportionately higher and the incidence is greater in LMIC than in HIC,^68^ the disaster response in HIC is unlikely to be externally valid in a low-income country setting. Despite this disparity, research from LMIC disaster response is under-represented in the literature.^1 68^

## Conclusion

EMT patient safety practices in disaster response is clinical-practice heavy and lacks many of the organisational and monitoring tools readily seen in HIC and non-disaster settings. Additionally, there does not appear to be a standardised method of medical record-keeping, although the importance of improving data capture is recognised by all quarters. As an extension of this, the ICPS framework is not easily adaptable to a disaster setting.

Based on our findings, we make the following recommendations: EMTs ideally under the auspices of an appropriate governing body, such as the WHO, should collaborate to achieve consensus on standardised and centralised medical record-keeping. EMTs should also strive for consensus on disaster zone-specific key indicators for safe practice, which should be incorporated within the medical record-keeping system. Patient safety practices should be encouraged by ensuring clinical teams are always supported by EMT managers who demonstrate safety leadership. The management of NCDs should be given greater representation in EMT missions. Where possible, patient safety practices should be evaluated within prospective trials.

We believe these actions will better inform EMT stakeholders of how safe their practice is and facilitate EMT standards setting and benchmarking. Only then can the vision of safe healthcare for everyone, everywhere be achieved.
