# Supplementary material for: The patient safety practices of emergency medical teams in disaster zones: a systematic analysis
Source: BMJ Glob Health. 2019 Nov 14;4(6):e001889. doi: 10.1136/bmjgh-2019-001889 (PMC6861101; doi:10.1136/bmjgh-2019-001889)
Supplement: Supplementary data [file bmjgh-2019-001889supp001.pdf]

Supplement 1

Table 5 – Frequency of Thematic Analysis Codes

CODE (order of frequency)	FREQUENCY	%
Limb injury	56	8.86
Medical records	34	5.38
Surgery - decision making	29	4.59
Medicines safety	28	4.43
Protocol	28	4.43
Surgery - technique	28	4.43
Infectious disease	21	3.32
Abdominal injury	15	2.37
Physiological monitoring	15	2.37
Team	15	2.37
Training	15	2.37
Fracture	14	2.22
Wound care	14	2.22
Amputation	13	2.06
Indicator	13	2.06
Institutional monitoring	13	2.06
Capacity	12	1.9
Antenatal and maternal health	11	1.74
Recommended essentials list	11	1.74
Transfer/referral	11	1.74
Anaesthesia	10	1.58
Paediatrics	10	1.58
Surgery - planning	10	1.58
Casts/splints/traction/frames	9	1.42
Blood product safety	8	1.27
Burns	8	1.27
Ophthalmology	8	1.27
ICU	7	1.11
Rehabilitation	7	1.11
Surgery - device	7	1.11
Immunisation	6	0.95
Radiology	6	0.95
Antibiotics	5	0.79
Blast injury	5	0.79
Consent	5	0.79
Dressings	5	0.79
Follow-up	5	0.79
Neuro-spinal	5	0.79
Peri-operative care	5	0.79
Physiotherapy	5	0.79

Skin graft / flaps	5	0.79
Vascular	5	0.79
Chest injury	4	0.63
Device	4	0.63
Neck injury	4	0.63
Patient education	4	0.63
Airway	3	0.47
Bedsore	3	0.47
Crush injury	3	0.47
End of life care	3	0.47
General patient care	3	0.47
Head injury	3	0.47
Nursing	3	0.47
Nutrition	3	0.47
Patient safety culture	3	0.47
Scoring system	3	0.47
VTE prophylaxis	3	0.47
Ward round /review	3	0.47
Cold injury	2	0.32
Communication	2	0.32
Counselling	2	0.32
Debrief	2	0.32
Diagnostic	2	0.32
HIV	2	0.32
Max-fax	2	0.32
NCD	2	0.32
Positioning	2	0.32
Respiratory distress	2	0.32
Sexual and reproductive health	2	0.32
Supervision	2	0.32
Complaints management	1	0.16
Disposal	1	0.16
Gastric protection	1	0.16
Incident report	1	0.16
Iv fluid	1	0.16
Limb injury - hand	1	0.16
Mechanical ventilation	1	0.16
Mental health	1	0.16
Needle stick	1	0.16
Renal	1	0.16
Sterility	1	0.16
Surgery - operation note	1	0.16
Tracheostomy	1	0.16
Triage	1	0.16

Table 6 – 30 most frequent thematic clinical codes

CODE (clinical)	FREQUENCY (/407)	%
Limb injury	56	13.8
Surgery - decision making	29	7.13
Surgery - technique	28	6.88
Infectious disease	21	5.16
Abdominal injury	15	3.69
Physiological monitoring	15	3.69
Fracture	14	3.44
Wound care	14	3.44
Amputation	13	3.19
Antenatal and maternal health	11	2.7
Anaesthesia	10	2.46
Surgery - planning	10	2.46
Paediatrics	10	2.46
Casts/splints/traction/frames	9	2.21
Burns	8	1.97
Ophthalmology	8	1.97
Rehabilitation	7	1.72
Surgery - device	7	1.72
Intensive care unit	7	1.72
Radiology	6	1.47
Blast injury	5	1.23
Consent	5	1.23
Dressings	5	1.23
Follow-up	5	1.23
Neuro-spinal	5	1.23
Peri-operative care	5	1.23
Physiotherapy	5	1.23
Skin graft / flaps	5	1.23
Vascular	5	1.23
Chest injury	4	0.98

Table 7 – Non-Clinical Thematic Codes

CODE (non-clinical)	FREQUENCY (/174)	%
Medical records	34	19.54
Protocol	28	16.09
Team	15	8.621
Indicator	13	7.471
Institutional monitoring	13	7.471
Training	15	8.621
Capacity	12	6.897
Recommended essentials list	11	6.322
Transfer/referral	11	6.322
Device	4	2.299
Patient education	4	2.299
Communication	2	1.149
Debrief	2	1.149
Patient safety culture	3	1.724
Supervision	2	1.149
Complaints management	1	0.575
Disposal	1	0.575
Incident report	1	0.575
Sterility	1	0.575
Surgery - operation note	1	0.575

Table 8 – Medicines Safety Thematic Codes

CODE (Medicines Safety)	FREQUENCY (/51)	%
Medicines safety	28	54.9
Blood product safety	8	15.69
Antibiotics	5	9.804
Immunisation	6	11.76
VTE prophylaxis	3	5.882
IV fluid	1	1.961

Table 9 – Frequency of Indicators

INDICATOR	FREQUENCY
Medical records	7
Infectious disease	5
Capacity	3
Medicines safety	2
Protocol	2
Blood product	1
Fracture	1
Immunisation	1
Paediatrics	1
Recommended essentials list	1
Surgery	1
